# Genome-Wide Association and Genomic Prediction of Powdery Mildew and CYSDV Resistance in Melon

**DOI:** 10.3390/plants15193011

**Published:** 2026-09-30

**Authors:** Krishna Sai Karnatam, Roopa Sowjanya Potlannagari, Carlos Lopez-Ortiz, Padma Nimmakayala, Amnon Levi, Umesh K. Reddy

**Affiliations:** 1 Department of Biology, Gus R. Douglass Institute, West Virginia State University, Institute, WV 25112, USA; krishna.karnatam@wvstateu.edu (K.S.K.); roopa.potlannagari@wvstateu.edu (R.S.P.); carlos.ortiz@wvstateu.edu (C.L.-O.); padma@wvstateu.edu (P.N.); 2 U.S. Vegetable Laboratory, U.S. Department of Agriculture-Agricultural Research Service, Charleston, SC 29414, USA; amnon.levi@ars.usda.gov

**Keywords:** melon, cucurbit powdery mildew, cucurbit yellow stunting disorder virus, GWAS, GS

## Abstract

Cucurbit powdery mildew (CPM) and Cucurbit yellow stunting disorder virus (CYSDV) are major constraints to sustainable melon (*Cucumis melo* L.) production worldwide. To elucidate the genetic basis of resistance and accelerate breeding, we integrated genome-wide association studies (GWASs), transcriptome validation, and genomic selection (GS) using a global panel of 341 melon accessions. Re-analysis of GWAS using BLINK and FarmCPU identified 18 and 19 marker–trait associations exceeding the exploratory threshold for CPM and CYSDV, respectively. Candidate gene analysis highlighted genes involved in lipid metabolism, membrane integrity, hormone signaling, transcriptional regulation, reactive oxygen species detoxification, and antiviral defense, including MLO-like protein, lipid transfer protein, hydroxyproline-rich glycoprotein, glutathione synthetase, RNA helicase, and elongation factor-like protein. Transcriptome analysis further validated several CPM-associated loci through infection-responsive expression patterns, strengthening their candidacy for disease resistance. Genomic prediction using Bayesian Ridge (BRR), gBLUP, Bayesian LASSO, Bayes A, and Bayes B, evaluated under 10-fold cross-validation, achieved consistently high prediction accuracies for CPM and moderate accuracies for CYSDV, reflecting differences in their underlying genetic architecture. Collectively, these findings identify putative associated resistance loci and demonstrate the utility of integrating GWAS, transcriptomics, and genomic selection to enhance marker-assisted and genomic-assisted breeding for durable resistance to fungal and viral diseases in melon.

## 1. Introduction

Melon (*Cucumis melo* L.) is one of the most economically important horticultural crops belonging to the Cucurbitaceae family and is cultivated extensively in both tropical and temperate regions worldwide [1]. The species is widely distributed across Asia, Africa, Europe, and the Americas and exhibits remarkable adaptability to diverse agroecological conditions. Although *C. melo* is believed to have originated in Africa, its primary center of diversity extends across Asia, from Turkey to Japan [2]. Wild relatives of melon have been reported from several countries, including Australia, Indonesia, Nepal, Japan, India, China, Cape Verde, and Angola, reflecting the extensive geographical distribution and genetic diversity of the species. Owing to its extraordinary variation in fruit morphology, flavor, texture, color, shape, and size, melon is regarded as one of the most diverse species within the genus *Cucumis* [2,3]. Historically, [4] classified *C. melo* into ten botanical varieties based on systematic crossing studies, while later taxonomic revisions simplified the classification system to accommodate the extensive diversity observed within the species [5].

Melon fruits are highly valued for their nutritional attributes, pleasant aroma, refreshing taste, and commercial importance [6]. Global melon production exceeds 28 million tonnes annually, emphasizing its significance in the horticultural sector. China is the leading producer with more than 14 million tonnes annually, followed by Turkey and India, which contribute substantially to global production [7].

Despite its economic and nutritional importance, melon production is severely constrained by numerous biotic stresses, with fungal and viral diseases being particularly destructive. These diseases adversely affect plant growth, photosynthesis, fruit development, and overall productivity, resulting in significant yield and quality losses. Among them, cucurbit powdery mildew (CPM) and Cucurbit Yellow Stunting Disorder Virus (CYSDV) are considered major threats to melon cultivation worldwide [8]. Cucurbit powdery mildew is one of the most important diseases affecting cucurbit crops under open-field and greenhouse conditions [9]. In several melon-producing regions, including Argentina, CPM is regarded as the most prevalent disease of cucurbits [10]. The disease is characterized by the appearance of white, powdery fungal colonies consisting of mycelia and conidia on the upper and lower leaf surfaces, petioles, and stems [11]. Initial symptoms typically appear as small chlorotic or pale-yellow lesions on older leaves, which rapidly develop into powdery white fungal growth [12]. As infection progresses, affected tissues undergo chlorosis, necrosis, and premature senescence, substantially reducing photosynthetic capacity and plant vigor [13]. Although fruits are rarely infected directly, severe defoliation can reduce fruit size, lower sugar accumulation, cause uneven ripening, impair coloration, and increase susceptibility to sunscald, ultimately causing considerable economic losses. Furthermore, the ability of powdery mildew pathogens to develop and spread in the absence of free water enhances their epidemiological success and complicates disease management strategies [14].

Among viral diseases, Cucurbit Yellow Stunting Disorder Virus (CYSDV) has emerged as a major threat to cucurbit production worldwide. CYSDV belongs to the genus *Crinivirus* within the family Closteroviridae and primarily infects melon, watermelon, cucumber, and squash crops. The virus was first reported in southeastern Spain on melon and cucumber and has subsequently spread throughout Europe, North Africa, the Middle East, and North America. CYSDV has been detected in countries including Egypt, France, Israel, Jordan, Lebanon, Mexico, Morocco, Portugal, Saudi Arabia, Syria, Turkey, the United Arab Emirates, and the United States [15]. The virus is transmitted exclusively by the whitefly *Bemisia argentifolii*, including biotype B, whereas the greenhouse whitefly (*Trialeurodes vaporariorum*) is not known to transmit the pathogen efficiently. CYSDV is neither mechanically transmitted nor seed-borne, making whitefly-mediated transmission the primary mechanism of disease spread [16]. Long-distance dissemination occurs through the movement of infected transplants and viruliferous whiteflies, while symptomless infected plants contribute significantly to disease establishment in new production areas. Infected melon plants typically exhibit interveinal chlorosis, extensive leaf yellowing, reduced vigor, stunting, and diminished fruit development. These symptoms impair photosynthetic efficiency, resulting in reduced fruit size, lower sugar content, delayed maturation, and substantial yield losses [15].

The increasing incidence and geographical spread of both cucurbit powdery mildew and CYSDV pose significant challenges to sustainable melon production [15,17]. These diseases reduce yield and fruit quality and increase production costs associated with disease management and vector control [18]. Therefore, a comprehensive understanding of their biology, epidemiology, symptom development, and economic impact is essential for developing effective integrated disease management strategies. Advances in host plant resistance (HPR) breeding, pathogen surveillance, and vector management will play a critical role in mitigating the impact of these diseases and ensuring sustainable melon production under changing climatic and agricultural conditions. GWAS have become a powerful approach for dissecting the genetic architecture of complex traits in several cereal and vegetable crops by exploiting natural genetic variation at the whole-genome level [19,20,21]. GWAS has successfully identified genomic regions and candidate genes associated with resistance to major biotic stresses, including fungal, bacterial, and viral diseases, and insect pests, as well as tolerance to abiotic stresses such as drought, salinity, heat, and cold [1,22,23,24,25]. These discoveries have accelerated marker-assisted breeding, genomic selection, and functional genomics, facilitating the development of climate-resilient and disease-resistant crop cultivars. HPR-based breeding programs can reduce the amount of chemicals, such as fungicides and insecticides, needed to control pathogens and their vectors. In this research, we associated SNPs with CPM and CYSDV resistance, conducted transcriptome analysis to identify genes regulating disease-resistance pathways, and developed genomic selection models for disease-resistance breeding programs in melon. The associated SNPs can be used for MQTL analysis to dissect genomic regions responsible for multiple disease resistance in melon.

## 2. Results

### 2.1. Genotyping and Population Structure

A total of 26,261 and 11,610 SNPs were used for GWAS and population study and GS, respectively (Table S1). The SNPs were distributed across all 12 chromosomes, providing comprehensive genome-wide coverage (Table S2). For the GWAS dataset, the number of SNPs per chromosome ranged from 1595 (chrom 10) to 2749 (chrom 6). Chrom 6 (2749 SNPs), 4 (2642 SNPs), and 1 (2613 SNPs) contained the highest marker densities, whereas chrom 10 (1595 SNPs), 9 (1763 SNPs), and 12 (1876 SNPs) had comparatively fewer markers. Similarly, the GS marker set comprised 11,610 SNPs, with chromosome-wise SNP counts ranging from 645 (chrom 10) to 1598 (chrom 5). The highest number of markers was observed on chrom 5 (1598 SNPs), followed by chrom 1 (1539 SNPs) and chrom 4 (1264 SNPs), while chrom 10 (645 SNPs), 9 (704 SNPs), and 12 (730 SNPs) had the fewest markers. Linkage disequilibrium declined rapidly with increasing physical distance. The mean (r^2^) decreased from approximately 0.29 at very short marker distances to about 0.08 by 25 kb, indicating that most LD decay occurred within the first ~25 kb. Beyond 25 kb, (r^2^) remained relatively low and fluctuated generally between approximately 0.04 and 0.08 across longer genomic distances (Figure S1).

**Figure 1:**
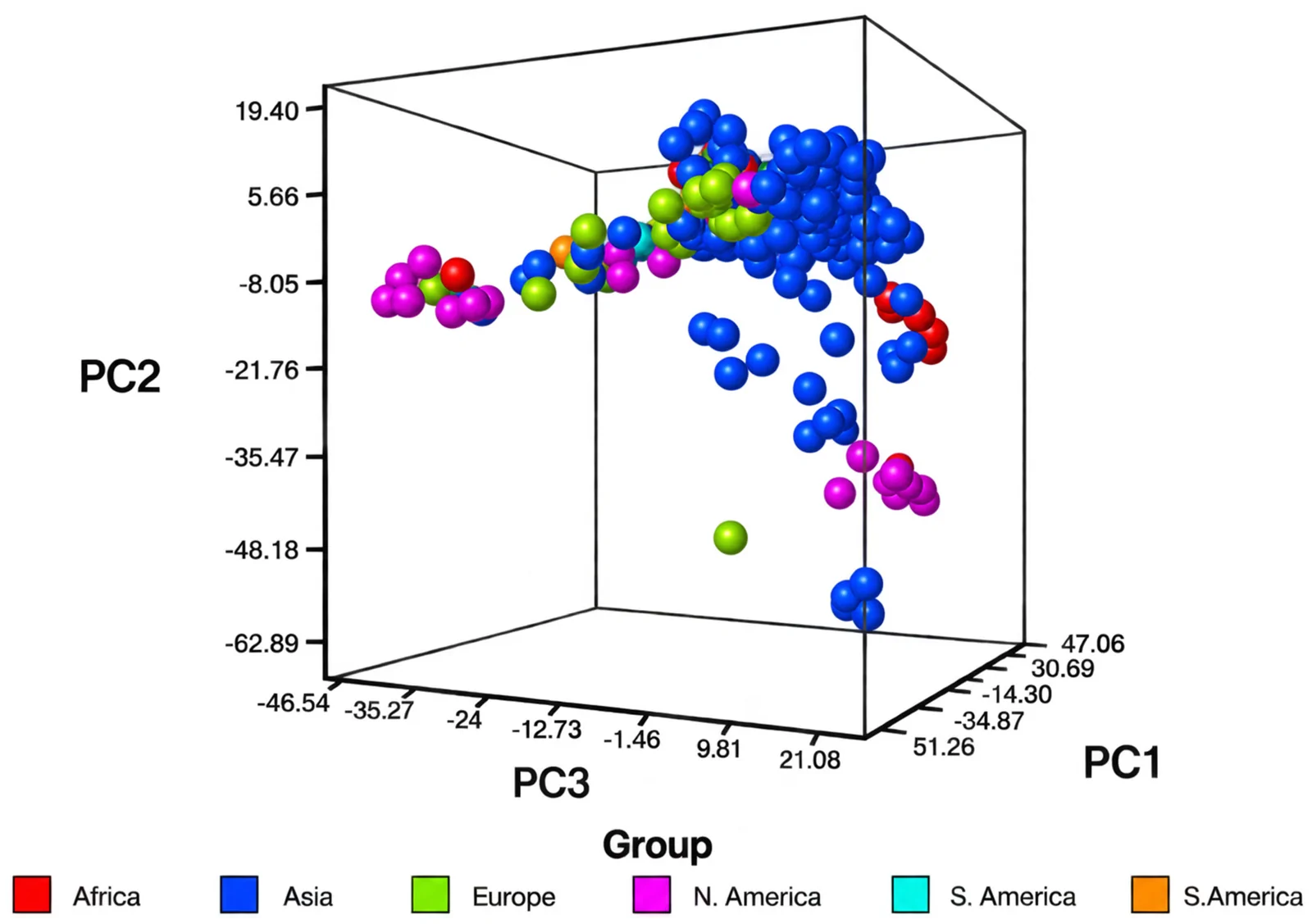
The 3D PCA of the melon core panel illustrates the underlying genetic structure of the population. Each colored dot represents a single accession and is colored by continent of origin: Africa (red), Asia (blue), Europe (green), North America (purple), South America (cyan), and Central America (orange).

Principal component analysis (PCA) was performed to investigate the genetic diversity and population structure of the melon germplasm panel based on genome-wide SNP markers. The 3D PCA revealed a continuous genetic distribution rather than distinct discrete clusters, indicating substantial genetic diversity with moderate population stratification among the accessions (Figure 1). The accessions were grouped by geographical origin (Africa, Asia, Europe, North America, and South America). Asian accessions constituted the largest proportion of the panel and were widely distributed across the PCA space, occupying almost the entire range of PC1, PC2, and PC3. The germplasm exhibits moderate population structure rather than strong stratification.

The Asian germplasm harbors the highest level of genetic diversity within the panel with multiple genetic backgrounds (Table S2). In contrast, European accessions formed a relatively compact cluster, indicating comparatively lower genetic variability and a narrower genetic base. North American accessions showed moderate clustering but were separated mainly by lower PC2 values, with several accessions extending toward PC1 values. Similarly, South American accessions were represented by only a few genotypes, which were scattered among the North American and Asian clusters without forming a distinct group, suggesting shared ancestry of the germplasm exchange. The African accessions were dispersed, with several individuals overlapping extensively with Asian germplasm, indicating close genetic relationships despite their different geographical origins (Figure 1).

Overall, considerable overlap was observed among accessions from different continents, particularly between Asia and Africa, and between North and South America. The absence of clearly separated geographic clusters indicates that geographical origin alone does not explain the observed genetic variation, likely reflecting extensive germplasm exchange, domestication history, breeding activities, and the movement of elite cultivars across continents. The presence of a few isolated accessions further suggests unique genetic backgrounds that could serve as valuable sources of novel alleles for breeding programs.

### 2.2. Disease Score Distribution in Population

The melon diversity panel exhibited phenotypic variation in resistance to CPM and CYSDV, indicating a broad spectrum of disease responses suitable for genome-wide association studies and genomic prediction.

**Figure 2:**
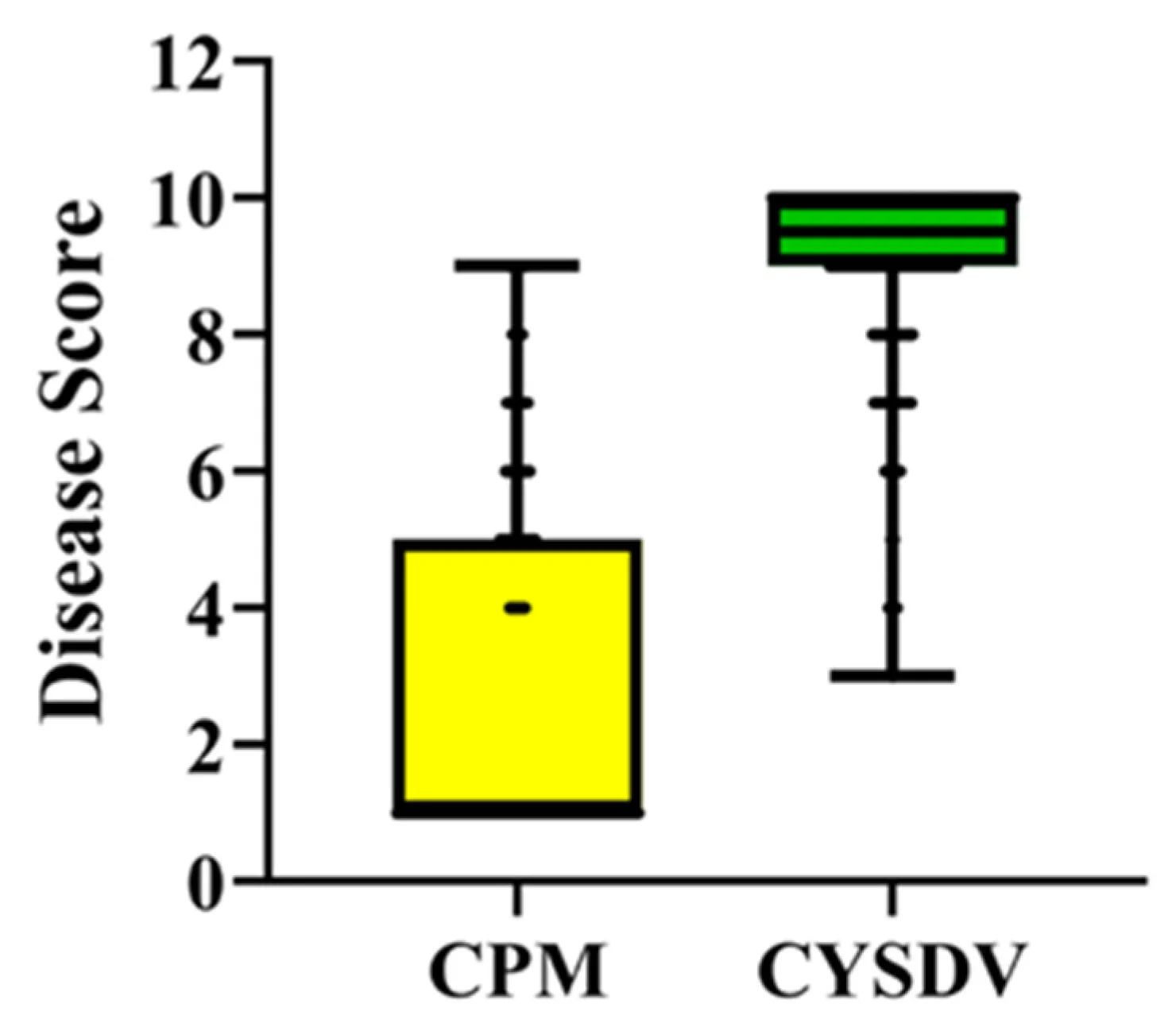
Boxplot showing the phenotypic distribution of disease scores for CPM and CYSDV across 341 melon accessions used in genome-wide association studies.

For CPM, disease scores ranged from 1 to 9, with most accessions scoring 1, indicating a predominance of highly resistant genotypes within the panel. A smaller proportion of accessions showed moderate susceptibility (scores 4–7), whereas only a few genotypes exhibited high susceptibility (scores 8–9). This skewed distribution toward lower disease scores suggests that resistance to CPM is relatively common in the germplasm collection, while highly susceptible genotypes are comparatively rare. In contrast, CYSDV scores ranged from 3 to 10, demonstrating a wider distribution of disease severity. Most accessions displayed intermediate to high disease scores (7–10), with a large proportion recording the maximum score of 10, indicating widespread susceptibility to CYSDV. Only a limited number of accessions showed relatively low disease scores (3–5), suggesting that strong resistance to CYSDV is less frequent than resistance to CPM (Figure 2 and Table S3).

To identify elite melon accessions with broad-spectrum resistance, genotypes were ranked based on their combined reactions to CPM and CYSDV (Table S1). Five accessions consistently performed best across both diseases. Ames_26703 (United States) ranked first, with the highest combined resistance (CPM score of 1 and CYSDV score of 3), indicating excellent resistance to both pathogens (Table 1). This accession represents the most promising source of multiple disease resistance for melon improvement.

The geographical distribution of resistant accessions showed that Indian germplasm contributed the most resistant or moderately resistant accessions, reflecting the extensive representation and genetic diversity of Indian melon collections in the panel. Resistant accessions were also identified from Afghanistan, Iran, Turkey, Spain, the United States, Egypt, Zimbabwe, Pakistan, and several other countries, demonstrating that useful resistance alleles are distributed across diverse geographical regions. However, susceptible genotypes were likewise observed across multiple origins, suggesting that geographical origin alone does not determine disease resistance. Overall, the wide phenotypic variation observed for both CPM and CYSDV provides a robust foundation for identifying genomic regions associated with disease resistance through GWAS and for developing accurate genomic prediction models.

The combined phenotypic assessment further revealed considerable variation in responses to the two diseases. Several accessions exhibited high resistance to CPM but moderate-to-high susceptibility to CYSDV, indicating that resistance to these diseases is likely governed by distinct genetic mechanisms. Conversely, a small number of accessions displayed relatively low disease scores for both pathogens, representing promising sources of broad-spectrum disease resistance for breeding programs.

### 2.3. GWAS and Candidate Gene Analysis for CPM and CYSDV

**Figure 3:**
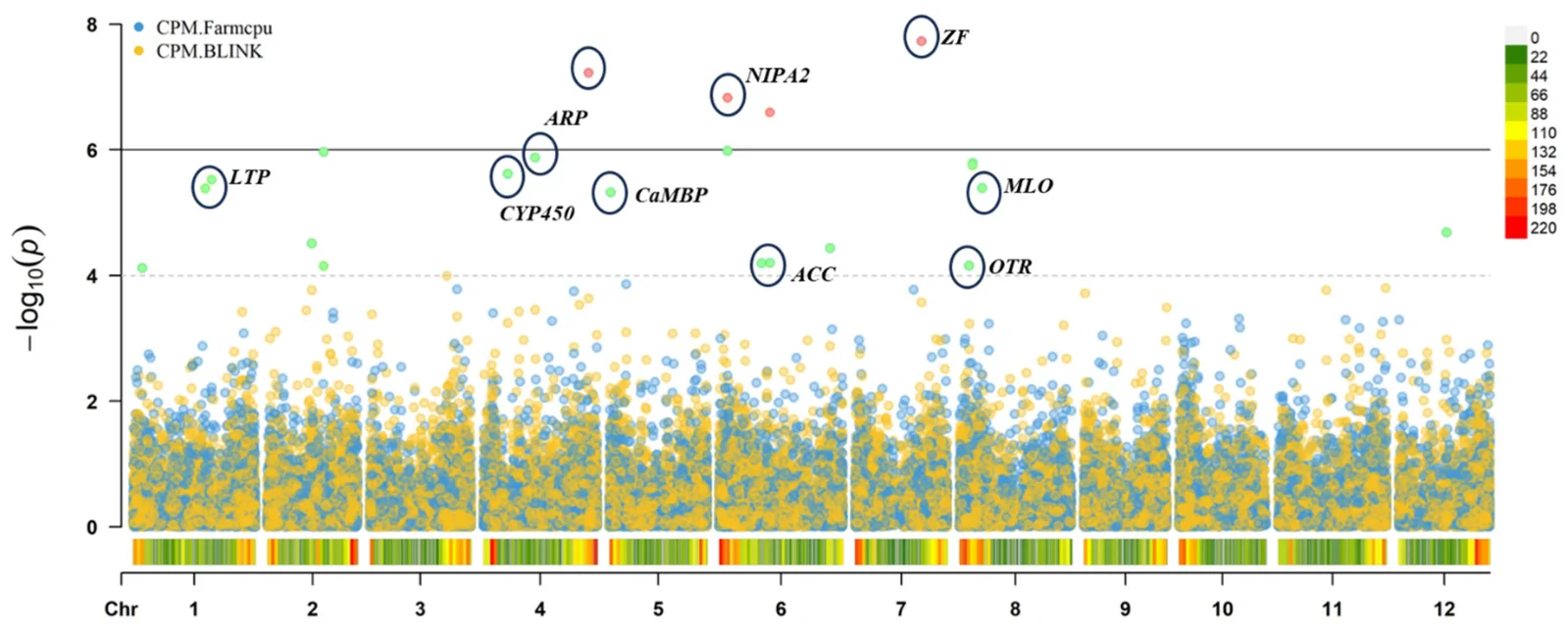
Manhattan plot of genome-wide association analysis for CPM resistance in melon using the BLINK (Blue dot) and FarmCPU (Yellow dot) models. The horizontal line indicates the significance threshold. Large open circles highlight SNPs associated with nearby candidate genes.

GWAS analysis for CPM identified 18 MTAs distributed across 8 chromosomes using the BLINK and FarmCPU models. Among these, FarmCPU detected 16 MTAs, whereas BLINK identified 6 SNPs, with four loci (S2_16309986, S6_2402534, S6_14765236, and S8_3767241) being consistently detected by both models (Table 2; Figure 3 and Figure S2). Chrom 6 harbored the highest number of associated loci (four SNPs), followed by chromosomes 1, 4, and 8 (three SNPs each), while chromosome 2 contained two SNPs and chromosomes 5, 7, and 12 each contained a single significant association. The detected loci exhibited strong statistical significance, with *p*-values ranging from 5.96 × 10^−8^ to 7.58 × 10^−5^. The most significant association was identified on chromosome 4 (S4_30546977; *p* = 5.96 × 10^−8^) by the FarmCPU model.

**Figure 4:**
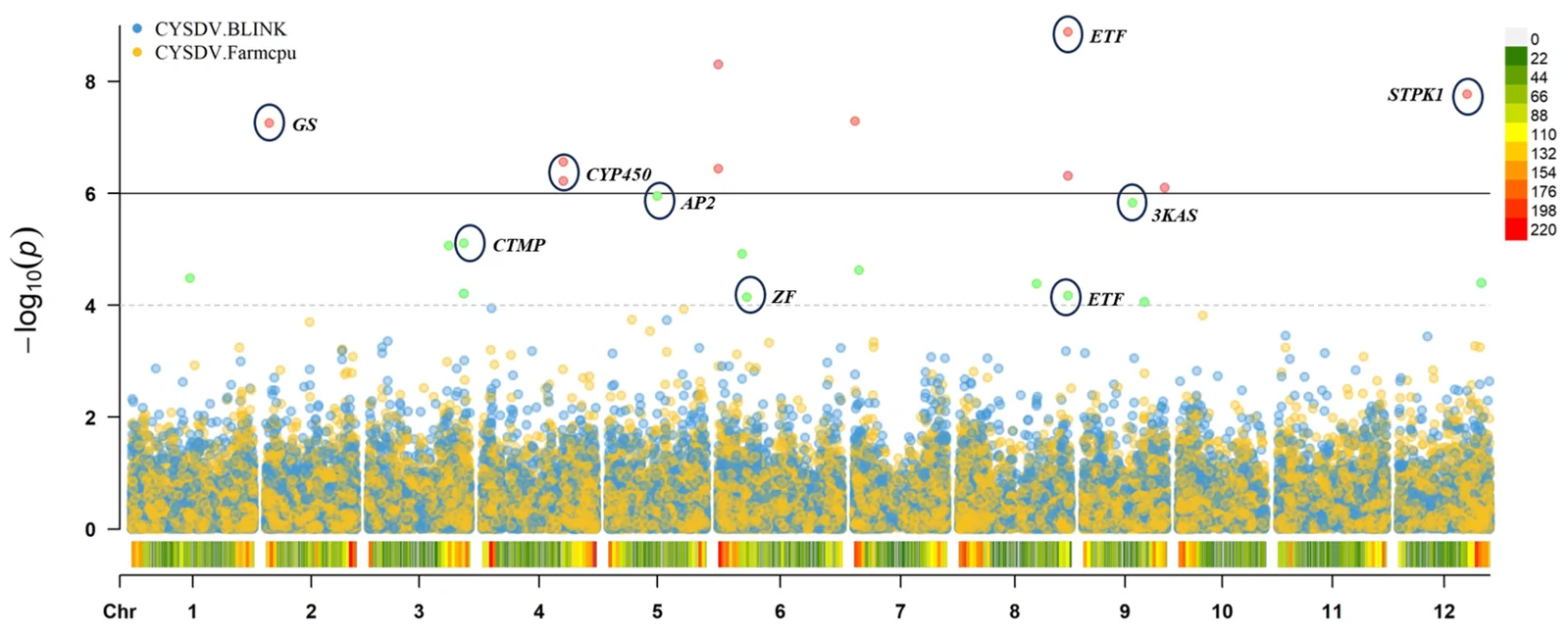
Manhattan plot of genome-wide association analysis for CYSDV resistance in melon using the BLINK (Blue dot) and FarmCPU (Yellow dot) models. The horizontal line indicates the significance threshold. Large open circles highlight SNPs associated with nearby candidate genes.

Genome-wide association analysis for CYSDV identified 19 significant marker–trait associations (MTAs) distributed across ten chromosomes (Chr1, 2, 3, 4, 5, 6, 7, 8, 9, and 12) using the BLINK and FarmCPU models (Table 2; Figure 4 and Figure S2). Among these, FarmCPU detected 18 SNPs, whereas BLINK identified 9 SNPs. Five loci, including S3_27607781, S4_23579056, S6_33816, S8_31658831, and S8_31654055, were identified by both models, representing putative genomic regions associated with CYSDV resistance.

The significant SNPs were unevenly distributed across the melon genome. Chromosomes 6 and 8 each contained four associated loci, followed by chromosomes 3 and 9 with three loci each, while chromosomes 1, 2, 4, 5, 7, and 12 each harbored one or two significant associations. The identified loci exhibited strong statistical significance, with *p*-values ranging from 4.99 × 10^−9^ to 8.76 × 10^−5^. The most significant association was detected on chromosome 8 (S8_31658831; *p* = 1.30 × 10^−9^) by the BLINK model, followed by loci on chromosome 6 (S6_33816; *p* = 4.99 × 10^−9^) and chromosome 12 (S12_19886385; *p* = 1.69 × 10^−8^) identified by FarmCPU.

Candidate gene analysis identified that CPM has several biologically relevant genes associated with significant SNPs, suggesting roles in membrane transport, lipid metabolism, signaling, transcriptional regulation, and stress responses. The identified candidate genes included those encoding a late embryogenesis abundant (LEA) hydroxyproline-rich glycoprotein (MELO3C018746), a lipid transfer protein (MELO3C012662), a lipase family protein (MELO3C012746), an ABC-2 type transporter (MELO3C010096), and an early nodulin-like protein 14 (MELO3C010323), indicating potential functions in membrane stability, lipid transport, lipid remodeling, and cellular signaling. Other notable candidates comprised a cytochrome P450 (MELO3C026491), an auxin-responsive protein (MELO3C012952), a hydroxyproline-rich glycoprotein (MELO3C009498), and a calmodulin-binding protein (MELO3C014686), suggesting roles in hormone signaling, secondary metabolism, cell wall organization, and calcium-mediated signaling. Additionally, genes encoding a magnesium transporter NIPA2-like protein (MELO3C006298), an acetyl-CoA carboxylase beta subunit (MELO3C019569), an S-adenosyl-L-methionine-dependent methyltransferase (MELO3C020246), zinc finger proteins (MELO3C014060 and MELO3C016115), an Ovate transcriptional repressor (MELO3C007422), a DUF247 domain-containing protein (MELO3C007579), an MLO-like protein (MELO3C007979), and a short-chain dehydrogenase/reductase (SDR) family protein (MELO3C025583) indicate coordinated regulation of mineral transport, fatty acid biosynthesis, methylation, transcriptional control, cell development, and redox metabolism underlying CPM (Table 2).

Overall, the GWAS identified several putative associated genomic regions associated with CYSDV resistance, with loci consistently detected by both BLINK and FarmCPU representing the most reliable associations. Candidate gene analysis revealed that the significant SNPs were located within or near genes involved in cellular transport, antioxidant defense, transcriptional regulation, RNA metabolism, protein turnover, lipid biosynthesis, ion homeostasis, and stress-responsive signaling. Notable candidate genes included a kinesin-like protein (MELO3C013420), glutathione synthetase (MELO3C015345), chloroplast thylakoid membrane protein (MELO3C010945), copper transporter (MELO3C011494), cytochrome P450 (MELO3C014945), AP2-like ethylene-responsive transcription factor (MELO3C008602), ATP-dependent RNA helicase (MELO3C005932), TraB family protein (MELO3C006871), zinc knuckle family protein (MELO3C006992), ring finger protein (MELO3C017047, aminopeptidase M1 (MELO3C016894), elongation factor-like protein (MELO3C003261), TBCC domain-containing protein 1 (MELO3C008901), CAAX amino-terminal protease (MELO3C005852), 3-ketoacyl-CoA synthase 17 (MELO3C025100), sodium/hydrogen exchanger 7 (MELO3C005228), serine/threonine protein kinase 1 (MELO3C002766), and anthranilate phosphoribosyltransferase (MELO3C002275). These genes are associated with intracellular transport, reactive oxygen species detoxification, photosynthetic maintenance, hormone signaling, antiviral defense, ubiquitin-mediated protein degradation, protein maturation, kinase-mediated signal transduction, and primary metabolism. The repeated detection of the elongation factor-like protein (MELO3C003261) by both GWAS models suggests that this genomic region represents a major candidate locus for CYSDV resistance because elongation factors are known to participate in host–virus interactions and viral replication. Collectively, these findings provide valuable candidate genes for functional validation and marker-assisted breeding to improve CYSDV resistance in melon.

### 2.4. Previous GWAS Findings Reveal Confirmed and Novel Associations

Comparison of the present GWAS results with previously published results showed both common and additional genomic regions associated with CPM and CYSDV resistance in melon. For CPM resistance, the present study identified 18 MTAs distributed across chromosomes 1, 2, 4, 5, 6, 7, 8 and 12, while the published study reported one significant SNP on chromosome 8. Three SNPs from the present study were also located on chromosome 8, and the closest marker, S8_2702894, was approximately 1.07 Mb from the previously reported SNP. For CYSDV resistance, the present study identified 19 MTAs. Importantly, two SNPs, S6_33816 and S12_19886385, were located at exactly the same genomic positions as previously reported SNPs. These markers were associated with MELO3C005932, encoding an ATP-dependent RNA helicase, and MELO3C002766, encoding a serine/threonine protein kinase 1, respectively (Table 3).

### 2.5. Validation of GWAS Results Using Transcriptome Analysis

**Figure 5:**
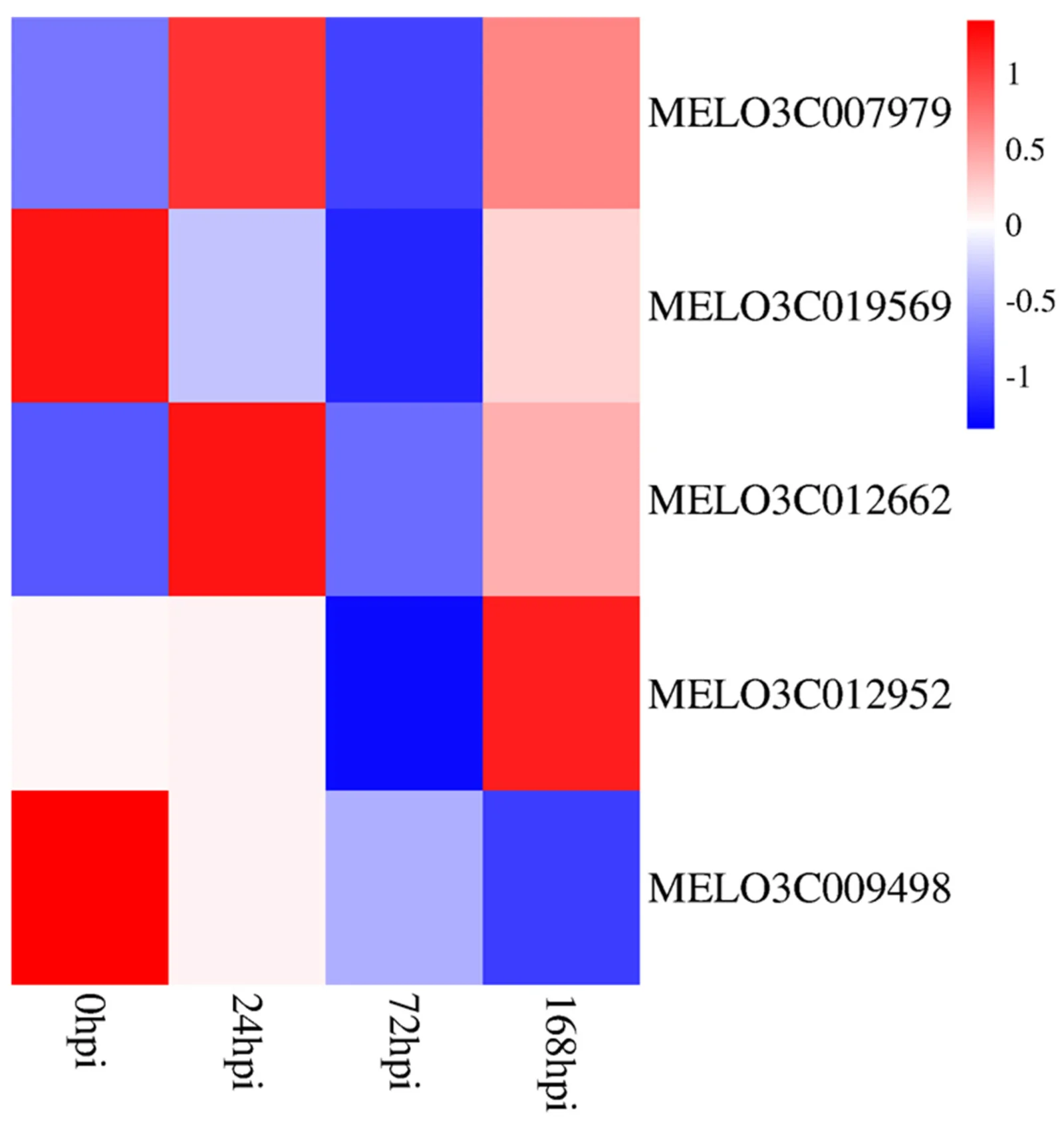
Expression patterns of representative GWAS candidate genes during Cucurbit Powdery Mildew infection in melon. Each column corresponds to a candidate gene, while rows denote expression at 0, 24, 72, and 168 h post-inoculation. Color intensity reflects relative transcript abundance (log_2_ fold change).

Expression profiling revealed marked differences in several GWAS-derived candidate genes relative to the corresponding reference group at each sampling time. The MLO-like gene (MELO3C007979) showed little expression difference at 24 h (log_2_FC = 0.03), followed by downregulation at 72 h (log_2_FC = −2.94) and 168 h (log_2_FC = −0.58). The lipid transfer protein gene (MELO3C012662) was upregulated at 24 h (log_2_FC = 3.09), followed by strong downregulation at 72 h (log_2_FC = −4.64), while no expression difference was detected at 168 h (log_2_FC = 0.00). The auxin-responsive gene (MELO3C012952) showed only a small expression difference at 24 h (log_2_FC = 0.26), followed by downregulation at 72 h (log_2_FC = −1.69) and upregulation at 168 h (log_2_FC = 1.86; approximately 3.64-fold higher expression). The hydroxyproline-rich glycoprotein gene (MELO3C009498) remained consistently upregulated at 24, 72, and 168 h, with log_2_FC values of 5.49, 4.78, and 3.91, respectively. Similarly, the acetyl-CoA carboxylase β-subunit gene (MELO3C019569) remained upregulated at 24, 72, and 168 h, with log_2_FC values of 2.50, 1.91, and 2.86, respectively (Figure 5 and Table S4).

### 2.6. GS for CPM and CYSDV

**Figure 6:**
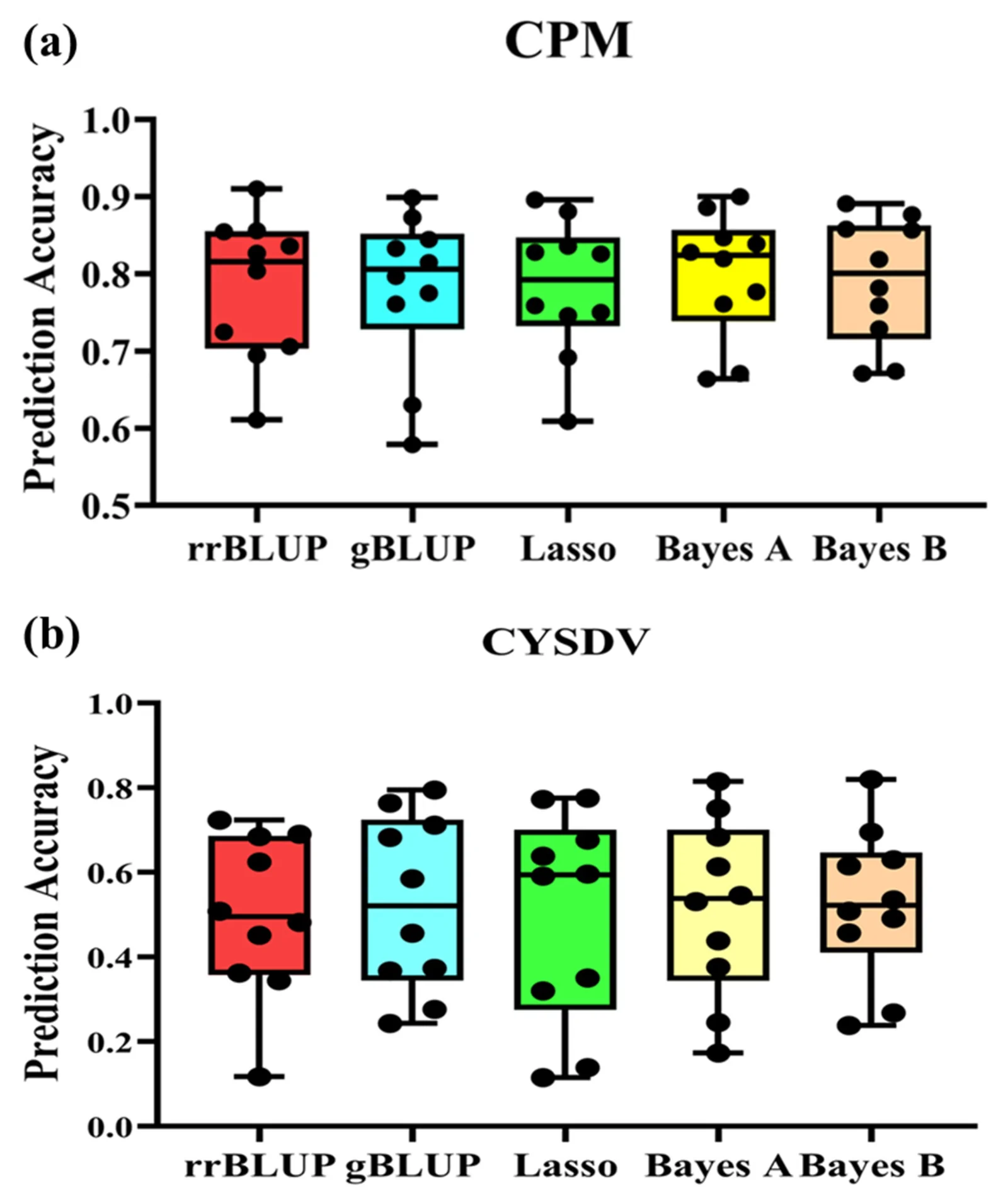
Comparison of genomic prediction accuracies for (**a**) CPM and (**b**) CYSDV resistance using different genomic selection models.

Five genomic selection models, Bayesian Ridge, gBLUP, LASSO, Bayes A, and Bayes B, were evaluated for predicting resistance to CPM and CYSDV using 10-fold cross-validation. All models demonstrated good predictive efficiency, with prediction accuracies ranging from 0.579 to 0.910 across validation runs. Among the evaluated models, Bayes A (0.799) produced the highest average prediction accuracy, followed closely by Bayes B (0.792), Bayesian Ridge (0.783), LASSO (0.782), and gBLUP (0.781). Although the mean prediction accuracies were comparable among models, differences were observed in their variability across cross-validation iterations. Bayesian Ridge exhibited consistently high prediction accuracy, reaching a maximum of 0.910, whereas gBLUP showed the greatest variation, with accuracies ranging from 0.579 to 0.899. LASSO achieved prediction accuracies between 0.609 and 0.896, while Bayes A and Bayes B reached maximum accuracies of 0.900 and 0.891, respectively. The distribution of prediction accuracies further indicated that all five models had median accuracies close to 0.80, with relatively narrow interquartile ranges (Figure 6), suggesting stable genomic prediction across repeated validation runs. Bayesian models generally exhibited slightly greater variability than Bayesian Ridge, whereas Bayesian Ridge maintained comparatively consistent performance across all cross-validation replicates.

Overall, the genomic prediction results demonstrated that CPM resistance was predicted with moderate to high accuracy (approximately 0.80) using genome-wide SNP markers. The comparable performance of Bayesian Ridge, gBLUP, LASSO, Bayes A, and Bayes B suggests that CPM is controlled by numerous loci with predominantly small-to-moderate genetic effects, indicating that genomic selection is a promising strategy for accelerating the breeding of CPM-resistant melon germplasm.

Similarly, the melon core was evaluated for CYSDV resistance using 10-fold cross-validation. Compared with CPM, the prediction accuracies for CYSDV were more variable among cross-validation iterations, indicating a relatively complex genetic architecture for the trait. Across all models, prediction accuracies ranged from 0.115 to 0.819. Among the evaluated models, Bayes A achieved the highest average prediction accuracy (0.517), followed closely by gBLUP (0.525), Bayes B (0.526), LASSO (0.497), and Bayesian Ridge (0.499). Although the average prediction accuracies of the five models were similar, differences were observed in their prediction stability. The highest single prediction accuracy (0.819) was obtained using Bayes B, followed by Bayes A (0.814) and gBLUP (0.794). LASSO achieved a maximum prediction accuracy of 0.775, whereas Bayesian Ridge reached 0.723. The distribution of prediction accuracies showed that all models had median values ranging from approximately 0.50 to 0.60.

Among the tested models, gBLUP and Bayes B exhibited relatively more stable prediction performance, whereas Bayesian Ridge and LASSO showed greater fluctuations between validation runs. Overall, genomic prediction accuracies for CYSDV were moderate, with average prediction accuracies of approximately 0.50 across all evaluated models. The comparable performance of Bayesian Ridge, gBLUP, LASSO, Bayes A, and Bayes B suggests that no single model provided a substantial advantage for predicting CYSDV resistance. Nevertheless, the moderate prediction accuracies demonstrate that genome-wide SNP markers capture a considerable proportion of the genetic variation underlying CYSDV resistance, highlighting the potential utility of genomic selection for accelerating breeding of resistant melon germplasm.

## 3. Discussion

Melon (*Cucumis melo* L.) is an economically important horticultural crop cultivated worldwide for its high nutritional and commercial value. However, its productivity and fruit quality are severely constrained by CPM and CYSDV, two of the most destructive diseases affecting melon production [15,26]. CPM causes premature leaf senescence and reduced photosynthetic efficiency, whereas CYSDV induces severe leaf chlorosis, stunting, and substantial yield losses. Chemical control of these diseases is often ineffective, costly, and environmentally unsustainable, particularly due to the rapid evolution of pathogen populations and insect vectors [17,27]. Consequently, developing disease-resistant cultivars through genomics-assisted breeding has become the most sustainable and economically viable strategy for achieving durable resistance and ensuring stable melon production [28]. In the present study, we integrated GWAS, transcriptome-based candidate gene validation, and genomic selection to dissect the genetic basis of resistance to CPM and CYSDV in melon.

The phenotypic distribution of disease scores revealed contrasting patterns between CPM and CYSDV. For CPM, disease scores showed a continuous distribution (1 to 9), which ranged from highly resistant to highly susceptible, suggesting that CPM resistance was relatively common in the panel. In contrast, CYSDV scores ranged from 3 to 10, with most accessions showing intermediate to high disease severity, indicating that strong CYSDV resistance was less frequent. The presence of some accessions with relatively low scores for both diseases is particularly important, as these genotypes may serve as useful donor lines for developing melon cultivars with combined resistance.

Candidate genes near CPM-associated SNPs were involved in lipid metabolism, membrane transport, cell wall organization, hormone signaling, transcriptional regulation, and stress responses. In *Arabidopsis thaliana*, GPI-anchored lipid transfer proteins (LTPGs) enhance pre-invasive immunity by facilitating cuticular lipid deposition and strengthening the epidermal barrier, thereby restricting fungal penetration; disruption of these genes results in increased susceptibility to *Blumeria graminis* [29]. Hydroxyproline-rich glycoproteins are key structural components of the plant cell wall and have been implicated in resistance to pathogen infection by strengthening cell walls through oxidative cross-linking. In pearl millet, this gene is responsible for enhanced resistance to *Sclerospora graminicola*, where increased hydroxyproline content and H_2_O_2_-dependent cross-linking reinforced cell walls and restricted pathogen invasion [30]. Cytochrome P450 enzymes have been widely implicated in plant defense by regulating the biosynthesis of secondary metabolites involved in pathogen resistance. In *Arabidopsis thaliana*, mutation of CYP83A1 enhanced resistance to powdery mildew by promoting camalexin accumulation and activating NPR1-, EDS1-, and PAD4-dependent defense pathways, highlighting the importance of cytochrome P450-mediated secondary metabolism in restricting fungal infection [31].

The identification of an MLO-like gene among CPM-associated candidate genes is especially noteworthy. In several plant species, MLO genes are well known for their role in powdery mildew susceptibility, and loss-of-function variation in specific MLO homologs can confer durable resistance. MLO (Mildew Locus O) genes are well-established regulators of plant susceptibility to powdery mildew, and loss-of-function mutations in these genes have been shown to confer durable and broad-spectrum resistance in several crop species, including barley, tomato, pea, cucumber, and melon. MLO proteins function as negative regulators of plant immunity by facilitating fungal penetration into epidermal cells, whereas disruption of MLO genes enhances pre-invasive defense through increased papilla formation and cell wall reinforcement [32].

The transcriptome-based approach further supported several CPM-associated candidate genes. Most GWAS-derived candidate genes exhibited differential expression during disease progression, indicating that the associated loci are transcriptionally responsive to infection. In particular, MELO3C009498, which encodes a hydroxyproline-rich glycoprotein family protein, and MELO3C007979, which encodes an MLO-like protein, were strongly induced during the early stage of infection. Integrating GWAS and expression data strengthens confidence in these genes as putative regulators of CPM resistance because genes that are both genetically associated with the trait and differentially expressed after infection are more likely to be functionally relevant.

For CYSDV resistance, candidate genes such as RNA helicases are involved in RNA metabolism, viral RNA processing, and host antiviral defense mechanisms [33]. In addition, translation elongation factors have been widely recognized as key host factors exploited by plant viruses during infection. Previous studies have demonstrated that eukaryotic translation elongation factors, particularly eEF1A, facilitate multiple stages of the viral life cycle, including viral RNA replication, translation, assembly, and pathogenesis, by interacting with viral replication complexes [34]. Elongation factors are known to participate in protein synthesis and may also influence host–virus interactions. Several other CYSDV candidate genes are consistent with known biological responses to viral infection. The glutathione synthetase gene may contribute to antioxidant defense and the detoxification of reactive oxygen species, both of which are commonly activated during virus-induced stress [35]. Overall, CYSDV resistance may involve antiviral defense, stress signaling, metabolic regulation, and cellular homeostasis. The recovery of two previously reported CYSDV-associated SNP positions further supports the consistency of the GWAS results, while the associated RNA helicase (MELO3C005932) and protein kinase (MELO3C002766) genes represent biologically relevant candidates for further validation. For CPM, a larger number of associations were detected than previously reported. Although the nearest chromosome 8 marker was approximately 1.07 Mb from the earlier SNP, this regional agreement suggests a potential role of chromosome 8 in CPM resistance. Additional loci identified on other chromosomes may reflect differences in germplasm, marker density, phenotyping, allele frequency, population structure, or GWAS models.

GS has emerged as an efficient breeding strategy for improving quantitatively inherited traits by capturing the cumulative effects of genome-wide markers, particularly for complex disease resistance traits controlled by numerous loci with small individual effects [36]. Previous studies in melon and other crop species have demonstrated that genomic prediction models, including Bayesian Ridge, GBLUP, Bayesian LASSO, Bayes A, and Bayes B, provide reliable prediction accuracies for disease resistance and can substantially accelerate breeding by reducing reliance on extensive phenotyping [37]. In the present study, CPM exhibited consistently high prediction accuracies across all genomic prediction models, suggesting that the genome-wide marker set effectively captured the genetic architecture underlying resistance.

In contrast, the prediction accuracy for CYSDV was moderate. Similar reductions in genomic prediction accuracy have been reported for disease resistance traits with lower heritability, stronger genotype-by-environment interactions, or limited representation of resistant alleles in the training population [37]. The greater variability among cross-validation replicates further indicates that prediction performance may be influenced by the composition of the training population and the distribution of resistant genotypes, emphasizing the importance of optimizing training population design for genomic selection in CYSDV resistance breeding.

In this study, the identification of putative associated GWAS loci together with expression-supported candidate genes provides valuable targets for marker-assisted selection, while genomic prediction offers an efficient approach for selecting superior genotypes carrying favorable allelic combinations [37,38]. Therefore, genomic selection appears particularly promising for accelerating improvements in CPM resistance, whereas breeding for CYSDV resistance may benefit from integrating genomic prediction with validated GWAS markers, resistant donor lines, and multi-environment phenotypic evaluations. Furthermore, the few accessions exhibiting resistance to both CPM and CYSDV represent valuable genetic resources for developing cultivars with broad-spectrum disease resistance.

## 4. Materials and Methods

### 4.1. Plant Material, Phenotypic Data Collection, Genotyping and Population Structure

A core set of 341 melon accessions, representing global melon germplasm diversity, was used to assess genetic variation underlying resistance to cucurbit powdery mildew (CPM) and cucurbit yellow stunting disorder virus (CYSDV). Disease scores for CPM and CYSDV resistance, generated from the 2018 screening experiment from a single environment, non-replicated field planting at the University of California Desert Research and Extension Center, Holtville, CA, in 2018, were obtained from [39], while SNP genotype data for the same melon core collection were retrieved from the Cucurbit Genomics Database (CuGenDB) [40]. The Melon reference genome DHL92 v3.5.1 was used in the genotypic file submitted in the CuGenDB [41]. SNP imputation was performed using a nearest-neighbor algorithm within small SNP windows, permitting 5% mismatch. A total of 26,261 high-quality SNPs that passed the quality control filtration criteria of call rate (CR) ≥ 0.7 to account for missing data and minor allele frequency (MAF ≥ 0.05) were used in GWAS analysis for CPM and CYSDV resistance [42]. The population structure was assessed using Principal Component Analysis (PCA) from GAPIT v3.0, as in [20,21]. The principal components were visualized using a 3D PCA tool from “Altius Institute Tools”. Population-wide linkage disequilibrium (LD) decay was estimated using PopLDdecay v3.45 by calculating pairwise LD (r^2^) between SNP markers as a function of physical distance [43].

### 4.2. GWAS and Candidate Gene Analysis

The GWAS for CPM and CYSDV was carried out using 26,261 high-quality SNPs with advance models BLINK and FarmCPU in the GAPIT v3.0 [44]. In this study, we utilized an exploratory threshold of − log10(*p*) = 4.00 to identify any SNP as statistically significant for disease resistance [45]. We considered a Bonferroni correction for 26,261 markers with a −log_10_(*p*) = 5.72. The SNPs were aligned from the melon reference genome v3.5.1 [41]. Gene annotation of candidate genes associated with GWAS-identified variation in disease resistance was performed using SnpEff, a widely used variant annotation and effect-prediction tool [21,46]. SnpEff locates SNPs within the reference genome and predicts their potential functional impact based on predicted coding and regulatory effects, thereby providing insight into the molecular mechanisms underlying variation in CPM resistance [47].

### 4.3. Validation of GWAS Analysis Using Transcriptome Analysis

The candidate genes identified through GWAS were further evaluated using the gene expression tool available in the CuGenDB. Expression values were obtained from the RNA-seq project PRJNA358655, which examined the responses of the contrasting melon genotypes MR-1 and Top Mark to powdery mildew (PM) stress using the Melon (DHL92) v.3.5.1 reference genome [48]. The dataset revealed both shared and genotype-specific gene-expression patterns between MR-1 and Top Mark under PM stress. These expression profiles were integrated with the positions of significant GWAS SNPs to prioritize promising candidate genes associated with CPM resistance [49,50]. This integrated analysis may provide new insights into the molecular mechanisms underlying CPM resistance in melon.

### 4.4. Genomic Selection for CPM and CYSDV Resistance

For genomic selection (GS), the SNP dataset was first subjected to marker quality filtering. Monomorphic markers and SNPs with missing genotype calls were removed, resulting in a final set of 11,610 high-quality SNPs [51]. The retained markers were converted into numerical genotype codes and used as the marker matrix for genomic prediction analysis. Genomic prediction was conducted using Bayesian and mixed-model approaches implemented in the BGLR-R v1.6 [52]. Genomic prediction was performed using five genomic selection models implemented in the BGLR-R software, including Bayesian Ridge, GBLUP, Bayesian LASSO, Bayes A, and Bayes B, to evaluate their predictive performance for disease resistance. Prediction performance was assessed using 10-fold cross-validation. The population was randomly divided into ten subsets, with nine subsets used for model training and the remaining subset used for validation in each fold. This process was repeated until all subsets had been used once as the validation set. Predictive ability was calculated as the correlation between observed phenotypic values and predicted genomic values, and prediction error was estimated using root mean square error.

## 5. Conclusions

This study identified genomic regions associated with CPM and CYSDV resistance in melon and prioritized putative candidate genes for further investigation. Genomic prediction results supported the potential application of genomic selection, particularly for CPM resistance, although evaluation in independent populations is needed to establish its breeding utility. The combined GWAS, transcriptome-based expression analysis, and genomic prediction results provide a basis for candidate prioritization and further research on disease resistance. Future studies should investigate candidate-gene functions through allele mining and functional analyses and evaluate associated markers and prediction models in independent populations. Selected candidates, including the MLO-like gene associated with CPM and the elongation factor candidate associated with CYSDV, could be investigated using approaches such as CRISPR-Cas9 to assess their roles in disease response and potential applications in melon breeding.

## Figures and Tables

**Table 1:** Top five melon accessions exhibiting the highest combined resistance to CPM and CYSDV based on disease scores and overall ranking.

Rank	Genotype	CPM Score	CYSDV Score
1	Ames_26703	1	3
2	Ames_13272	1	4
3	PI_116667	1	4
4	PI_536472	1	4
5	CPPSIH_4_02	1	5

**Table 2:** SNP Associations and Candidate Genes Identified for CPM and CYSDV Resistance in Melon.

SNP Position	Chrom	−log_10_(*p*)	Gene ID	Gene Function	Model	Trait
S1_2554116	1	4.12	MELO3C018746	Late embryogenesis abundant (LEA) hydroxyproline-rich glycoprotein family	FarmCPU	CPM
S1_20889109	1	5.38	MELO3C012662	Lipid transfer protein (LTP)	BLINK	CPM
S1_22776398	1	5.52	MELO3C012746	Lipase family protein (LFP)	BLINK	CPM
S2_12829291	2	4.51	MELO3C010096	ABC-2 type transporter family protein	FarmCPU	CPM
S2_16309986	2	4.15	MELO3C010323	Early nodulin-like protein 14, putative	FarmCPU, BLINK	CPM
S4_7101590	4	5.62	MELO3C026491	Cytochrome P450 (CYP450)	FarmCPU	CPM
S4_15115814	4	5.88	MELO3C012952	Auxin-responsive protein (ARP)	FarmCPU	CPM
S4_30546977	4	7.22	MELO3C009498	Hydroxyproline-rich glycoprotein family protein (HRGP)	FarmCPU	CPM
S5_392639	5	5.32	MELO3C014686	Calmodulin binding protein, putative (CaMBP)	FarmCPU	CPM
S6_2402534	6	6.83	MELO3C006298	Magnesium transporter NIPA2-like protein (NIPA2)	FarmCPU, BLINK	CPM
S6_12253531	6	4.20	MELO3C019569	Acetyl-CoA carboxylase beta subunit (ACC)	FarmCPU	CPM
S6_14765236	6	6.59	MELO3C020246	S-adenosyl-L-methionine-dependent methyltransferases superfamily protein	FarmCPU, BLINK	CPM
S6_32223011	6	4.43	MELO3C014060	Zinc finger protein 6 (ZF)	FarmCPU	CPM
S7_19277759	7	7.73	MELO3C016115	B-box zinc finger family protein (ZF)	FarmCPU	CPM
S8_2702894	8	4.16	MELO3C007422	Ovate transcriptional repressor (OTR)	FarmCPU	CPM
S8_3767241	8	5.75	MELO3C007579	Plant protein of unknown function (DUF247)	FarmCPU, BLINK	CPM
S8_6565244	8	5.39	MELO3C007979	MLO-like protein (MLO)	FarmCPU	CPM
S12_13826345	12	4.69	MELO3C025583	Dehydrogenase/reductase SDR family member 4	FarmCPU	CPM
S1_16913086	1	4.48	MELO3C013420	Kinesin-like protein	FarmCPU	CYSDV
S2_939031	2	7.25	MELO3C015345	Glutathione synthetase (GS)	FarmCPU	CYSDV
S3_27607781	3	5.11	MELO3C010945	Chloroplast thylakoid membrane (CTMP)	FarmCPU, BLINK	CYSDV
S3_23316898	3	5.06	MELO3C011494	Copper transporter	FarmCPU	CYSDV
S4_23579056	4	6.56	MELO3C014945	Cytochrome P450, putative (CYP450)	FarmCPU, BLINK	CYSDV
S5_14222070	5	5.95	MELO3C008602	AP2-like ethylene-responsive transcription factor (AP2)	FarmCPU	CYSDV
S6_33816	6	6.44	MELO3C005932	ATP-dependent RNA helicase, putative	FarmCPU, BLINK	CYSDV
S6_6876821	6	4.91	MELO3C006871	TraB family protein	FarmCPU	CYSDV
S6_8269942	6	4.14	MELO3C006992	Zinc knuckle family protein (ZF)	FarmCPU	CYSDV
S7_237628	7	7.29	MELO3C017047	Ring finger protein, putative	FarmCPU	CYSDV
S7_1374085	7	4.63	MELO3C016894	Aminopeptidase M1	FarmCPU	CYSDV
S8_31658831	8	8.89	MELO3C003261	Elongation factor-like protein (ETF)	FarmCPU, BLINK	CYSDV
S8_22531968	8	4.38	MELO3C008901	TBCC domain-containing protein 1	FarmCPU	CYSDV
S8_31654055	8	4.17	MELO3C003261	Elongation factor-like protein (ETF)	FarmCPU	CYSDV
S9_23635329	9	6.10	MELO3C005852	CAAX amino-terminal protease family	BLINK	CYSDV
S9_14307596	9	5.83	MELO3C025100	3-ketoacyl-CoA synthase 17 (3KAS)	FarmCPU	CYSDV
S9_17688379	9	4.06	MELO3C005228	Sodium/hydrogen exchanger 7	BLINK	CYSDV
S12_19886385	12	7.77	MELO3C002766	Serine/threonine protein kinase 1 (STPK1)	FarmCPU	CYSDV
S12_24006232	12	4.40	MELO3C002275	Anthranilate phosphoribosyltransferase	BLINK	CYSDV

**Table 3:** Comparison of Current GWAS Associations with Previously Reported Loci for CPM and CYSDV Resistance in Melon.

Trait	Present SNP	Candidate Gene	Published SNP Position	Distance	Position of the SNPs
CYSDV	S6_33816	MELO3C005932 ATP-dependent RNA helicase	Chr6:33,816	0 bp	Exact positional match
CYSDV	S12_19886385	MELO3C002766 Serine/threonine protein kinase 1	Chr12:19,886,385	0 bp	Exact positional match
CYSDV	S3_27607781	MELO3C010945 Chloroplast thylakoid membrane protein	Chr3:26,579,942	~1.03 Mb	Nearby region
CYSDV	S6_6876821	MELO3C006871 TraB family protein	Chr6:5,635,767	~1.24 Mb	Nearby region
CPM	S8_2702894	MELO3C007422 Ovate transcriptional repressor	Chr8:1,632,997	~1.07 Mb	Closest CPM association
CPM	S8_3767241	MELO3C007579 DUF247 protein	Chr8:1,632,997	~2.13 Mb	Same chromosome region
CPM	S8_6565244	MELO3C007979 MLO-like protein	Chr8:1,632,997	~4.93 Mb	Same chromosome; more distant

## Data Availability

The genotypic data were obtained from the Cucurbit Genomics Database (http://cucurbitgenomics.org, accessed on 11 June 2026), while the phenotypic data were obtained from the previously published study [34]. The transcriptomic data were retrieved from the NCBI Sequence Read Archive under BioProject accession PRJNA358655.

## Supplementary Materials

The following supporting information can be downloaded at: https://www.mdpi.com/article/10.3390/plants15193011/s1. Figure S1: Linkage disequilibrium (LD) decay in the melon population. LD was measured as the squared correlation coefficient (r^2^) between SNP markers across physical distance; Figure S2. Quantile–quantile (Q–Q) plots of observed versus expected −log_10_(*p*) values from genome-wide association analyses of disease resistance in 341 melon accessions: (a) CPM resistance using BLINK, (b) CPM resistance using FarmCPU, (c) CYSDV resistance using BLINK, and (d) CYSDV resistance using FarmCPU. Blue circles represent individual SNPs, the red line represents the expected null distribution, and the gray region indicates the 95% confidence interval. The upper-tail deviations indicate SNP associations exceeding the null expectation; Table S1: Chromosome-wise distribution of SNPs used for GWAS and GS analyses; Table S2: Genotypes used for GWAS and GS analyses, including their origin and subspecies; Table S3: Phenotypic disease scores for Cucurbit powdery mildew (CPM) and Cucurbit yellow stunting disorder virus (CYSDV) used for GWAS and GS analyses; Table S4: Expression profiles of GWAS-derived candidate genes associated with CPM resistance during pathogen infection at 0, 24, 72, and 168 h post-inoculation.
