# Supplementary material for: Genome-Wide Association and Genomic Prediction of Powdery Mildew and CYSDV Resistance in Melon
Source: Plants (Basel). 2026 Sep 30;15(19):3011. doi: 10.3390/plants15193011 (PMC13666691; doi:10.3390/plants15193011)
Supplement: Supplementary file 1 [file plants-15-03011-s001.zip › Supplementary Figures.pdf]

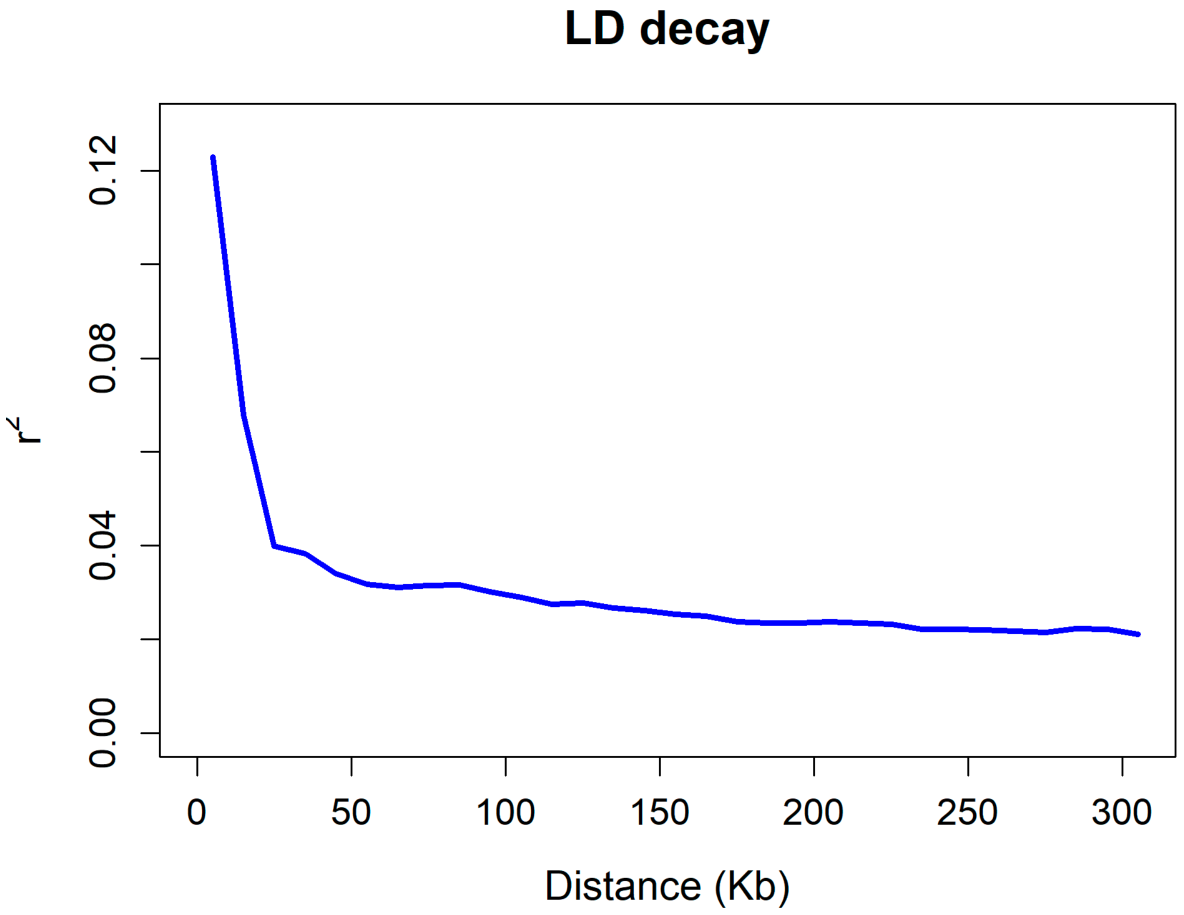

Figure S1. Linkage disequilibrium (LD) decay in the melon population. LD was measured as the squared correlation coefficient (r^2)) between SNP markers across physical distance.

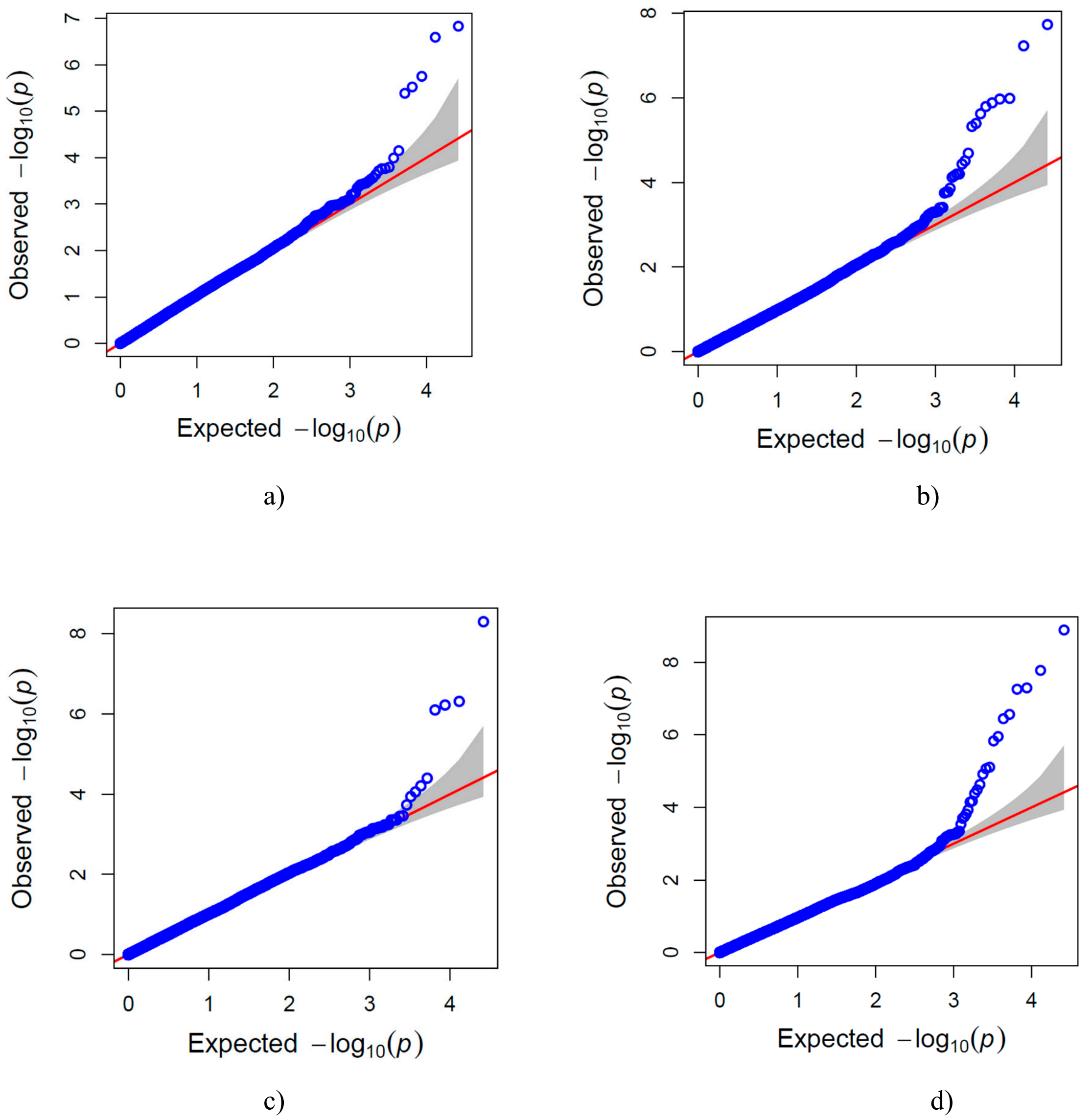

Figure S2. Quantile–quantile (Q–Q) plots of observed versus expected $-\log_{10}(P)$ values from genome-wide association analyses of disease resistance in 341 melon accessions: (a) CPM resistance using BLINK, (b) CPM resistance using FarmCPU, (c) CYSDV resistance using BLINK, and (d) CYSDV resistance using FarmCPU. Blue circles represent individual SNPs, the red line represents the expected null distribution, and the gray region indicates the 95% confidence interval. The upper-tail deviations indicate SNP associations exceeding the null expectation.
